# Drug‐facilitated sexual assault in Africa: A scoping review of empirical evidence

**DOI:** 10.1002/puh2.119

**Published:** 2023-09-08

**Authors:** Jimoh Amzat, Kehinde Kazeem Kanmodi, Kafayat Aminu, Eyinade Adeduntan Egbedina

**Affiliations:** ^1^ Department of Sociology Usmanu Danfodiyo University Sokoto Nigeria; ^2^ Department of Sociology University of Johannesburg Johannesburg South Africa; ^3^ School of Health and Life Sciences Teesside University Middlesbrough UK; ^4^ Cephas Health Research Initiative Inc Ibadan Nigeria; ^5^ Faculty of Dentistry University of Puthisastra Phnom Penh Cambodia; ^6^ School of Dentistry University of Rwanda Kigali Rwanda; ^7^ Centre for Child and Adolescent Mental Health University College Hospital Ibadan Nigeria

**Keywords:** Africa, drug‐facilitated sexual assault, nursing care, pastoral care, scoping review, sexual violence

## Abstract

**Background:**

Sexual assault has been a major social problem worldwide. Drug‐facilitated sexual assault (DFSA) is a form of sexual assault facilitated by psychoactive substances. DFSA is highly prevalent worldwide, though it is usually underreported. To understand the situation of DFSA in Africa, there is a need to map the available empirical evidence on DFSA in Africa. Hence, this scoping review was conducted to summarize the existing empirical knowledge and gaps in the literature and inform future research on DFSA in Africa.

**Methods:**

This study adopted the design by the Arksey and O'Malley's guideline for scoping reviews. Without year limiters, literatures were retrieved through a systematic search of the 11 electronic databases using appropriate search terms and Boolean operators. The retrieved literatures were deduplicated and screened, using Rayyan, to identify eligible literature for inclusion into the review. Only those articles that met the eligibility criteria were included for data charting, collation, and summarization. Four articles were included in this review.

**Results:**

Four articles were included in this review. The studies reported the characteristics of offenders and the context in which DFSA occurred. In the reviewed studies, rape of men was only reported among South Africans. Sedatives and alcohol are the most reported substances used in such rape. Perceptions of date rape, as documented by sexual assault survivors, suggest the pervasiveness of victim blaming. Typical DFSA cases appear opportunistic in nature, and an acquaintance is often the culprit. However, disaggregating DFSA as a specific sexual assault requires some logistics, including forensic and toxicological investigations.

**Conclusion:**

DFSA is a significant dimension of gender‐based sexual violence. Infrastructure development should be improved to support systematic toxicological analyses and services to investigate and understand DFSA.

## INTRODUCTION

Sexual assault has been a major social problem worldwide [1]. Unlike the concept of “rape,” “sexual assault” is broader and encompasses various dimensions of sexual offences. Rape is a forceful penetration of a woman by a male perpetrator [2]. This definition is regarded as gender‐biased as a female could also commit sexual offences. For the sake of gender neutrality and broader conception, the concept of “sexual assault” is adopted, which refers to all forms of nonconsensual sexual contact, including sexual fondling or unwanted sexual touching or kissing, and more importantly, any genital, oral, or anal penetration by a part of the perpetrator's body or by an object, using force or without the victim's consent [3]. Drug‐facilitated sexual assault (DFSA) is a form of sexual assault facilitated by psychoactive substances. It is an act of sexual violence toward a victim who is incapacitated due to the voluntary or involuntary consumption of intoxicating substances [4]. DFSA is highly prevalent worldwide, though it is usually underreported [5]. Low reportage is a common attribute of all sexual offences because sociocultural contexts could promote victim blame or stigma. Irrespective of toxicological analyses, DFSA must involve the use of certain substances to alter the mental or physical state of the victim [5].

It has been observed that the toxicological analysis of DFSA cases is particularly challenging when there is a time delay from assault to a medical examination [4]. This is why DFSA is under‐classified. Victims are hesitant to report incidents because of denigration, guilt, or self‐blame or because it is hard to remember the perpetrator (due to drug influence). Several drugs have been documented in DFSA, but alcohol is the most commonly detected substance and it co‐occurrs with other drugs [5]. Another complex dimension is that rapidly metabolized substances can be used in DFSA, making them not easily detectable in routine drug screenings [6]. However, most substances used (except alcohol) are under international control, whereas a few drugs (psychotropic substances and antihistamines), which are not under drug control, make investigation and classification a bit challenging [6].

Sexual assault is also a social problem and public health challenge in Africa [2, 7–9], with children and young girls remaining the most vulnerable [10]. One of the dangerous means and dimensions of sexual assault is perpetuated through drug facilitation. Hence, this study focuses on DFSA in Africa.

Several studies had been conducted on sexual health of different population groups in Africa. However, after a scoping search of multiple databases, it was observed that no known study has conducted a scoping review on DFSA in Africa. Hence, this is an effort to document the available evidence and interrogate how and what drugs are used in sexual assaults for the purpose of control. Hence, this study, a scoping review of empirical evidence on DFSA in Africa, aims to identify the existing knowledge and gaps in the literature and inform future research on this area of interest. The outcome of this review will provide insights that will help in prevention and control of DFSA in Africa.

## METHODS

### Design

This study was a scoping review of empirical evidence that seeks to identify the existing knowledge and gaps in the literature and inform future research on this area of interest [11]. This review was conducted and reported in accordance with the framework and guidelines for scoping reviews [12].

### Review question

This scoping review seeks to answer this question: What is the current empirical evidence on DFSAs in Africa?

### Literature selection

Only those literature on DFSAs in Africa that were empirical research articles (of any research design) published in peer‐reviewed journals, with accessible full text, and in English were included in this scoping review.

### Literature search

The literature search was done on December 30, 2022, with the aid of Boolean operators (“AND” and “OR”), using relevant Medical Subject Headings (MeSH) terms and synonyms (text words). Tables A1–A3 (Appendix) show the search strings generated from the search combinations used. The databases involved in the search were SCOPUS, PubMed, Allied and Complementary Medicine Database (AMED), CINAHL Complete, CINAHL Ultimate, Dentistry and Oral Sciences Source, MEDLINE, SPORTDiscus with Full Text, APA PsycArticles, APA PsycInfo, and Psychology and Behavioral Sciences Collection.

### Deduplication of literature

The obtained literature was retrieved and imported into the Rayyan web application for deduplication [13].

### Literature selection

With the aid of the Rayyan web application, a two‐stage blinded literature screening, performed by two independent researchers, was done to identify those deduplicated literatures relevant for inclusion in the scoping review [13]. The first stage of the screening process involved the review of titles and abstracts to exclude irrelevant literature at face value. Only the literatures included after the title and abstract screening were subjected to the second‐stage screening, which was a full‐text assessment. Only the articles that met the inclusion criteria were included in the scoping review.

### Data extraction, collation, and charting

From the included literature, data were extracted with the aid of a data extraction sheet customized for the scoping review. The extracted data include authors’ names, year of publication, research design, research objectives, the geographical location of the research, population under study, sample size, findings, and conclusions. The extracted data were then collated and summarized into themes and presented in texts and tables.

To determine the final themes presented in this review, thematic synthesis was used by one of the authors who carefully synthesized findings/data reported in the selected papers. The themes identified at the initial stage were assessed by another reviewer who was blinded to the data synthesis. The themes were then compared and re‐categorized to align with the focus of this review. Both reviewers later discussed the new set of themes and clarified areas of disagreement. After reaching a consensus, both reviewers agreed on the final set of themes.

## RESULTS

From the 11 electronic databases that were searched, only 92 hits (publications) were obtained. After deduplication, 12 publications were excluded. After the two‐stage publication screening, only four articles were finally included in this scoping review.

Four publications met the inclusion criteria; two of these were among Nigerians, and two among South Africans. Two studies used qualitative research methodology [8, 14], whereas two adopted a quantitative research approach [15, 16]. Three of the papers investigated the circumstances and experience of rape among survivors [8, 14, 16]; the fourth assessed knowledge and perception of date rape among female undergraduate students (including victims and non‐victims) [15]. Each of the studies among rape survivors interviewed men (and those identifying as men) and women, respectively. The review generated different themes on the topic of DFSA, such as awareness, knowledge and perception; incidence and prevalence of DFSA; common types of rape; patterns of DFSA and characteristics of assaulters; DFSA survivors’ relationship to abusers; forms of violence accompanying DFSA and reportage of DFSA. Additional information is depicted in Table 1. The major findings of this review include a high level of awareness about date rape and drugs being used to perpetrate the act. However, knowledge of the health implications of date rape on victims in the studies was low. Furthermore, the perceptions held in the studies were favorable to date rape as it was not perceived to be an “actual rape” unlike stranger rape. Another notable finding is the low, but rising reportage of rape cases. It was evident that victims lacked trust in law enforcement agents in Nigeria compared to South Africa. Sexual assault reportage was influenced by different factors, including stigma, accessibility of social support, and law enforcement agents. Likewise, the severity of physical assault inflicted, and injuries sustained by victims also determined whether sexual assault will be reported or not.

**TABLE 1:** Summary of studies on drug‐facilitated sexual assault (DFSA) included in the scoping review.

Authors and references	Objectives	Sample size and location	Drugs reportedly used or mentioned	Population	Age range	Study design	Key summary
Mgolozeli and Duma [8]	To determine and then describe, the types of rape experienced by men	11, Gauteng province, South Africa	Mostly alcohol with possible added sedative	Male survivors of rape	18–65 years	Interpretative phenomenological analysis (IPA) research design	It is important to provide post‐rape care for all rape survivors within the current post‐rape health‐care services
Oshiname et al. [15]	To assess undergraduate female students’ knowledge and perceptions of date rape	610, Ibadan, Nigeria	Alcohol and sleep‐inducing drugs; aphrodisiacs chemical/aerosols sprayed into the air	Undergraduate female students	17–30 years	Descriptive cross‐sectional survey	Students’ knowledge and their supportive perceptions about date rape can be modified through campus‐based educational activities
Aborisade [14]	To explore the patterns, nature, and factors responsible for the shift in sexual criminal behavior during the shutdown period (March and June 2020) and post‐assault behavior of the victims	25 (19 victims and 6 NGO officials), Lagos, Nigeria	Drugging as a means (drug not specified)	Female victims of sexual violence	13–37 years	Qualitative design	Women and child support networks are essential services during a lockdown
Tiemensma and Davies [16]	To preliminary investigate and characterize DFSA in a specific metropolitan setting in South Africa and to identify the drugs/xenobiotics associated with these reported drug facilitated sexual assaults	107 survivors of suspected DFSA, Cape Town, South Africa	Ethanol, methamphetamine, methaqualone, and diphenhydramine	107 survivors (female [*n* = 104, 97%])	All age categories but 18–25 years (*n* = 54, 50%)	Quantitative design	DFSA in this setting is mostly opportunistic, with ethanol suggested to be the most commonly involved drug There is a need for greater investment into the development of infrastructure to support systematic toxicological analyses and services to assist in the investigation and understanding of these intricate cases

Largely, sociocultural factors such as victim blaming, cultural beliefs, psychological cost of reporting rape, weak law enforcement system, and public health emergency enabled sexual assault as its incidence increased during COVID‐19 pandemic. Data synthesis also shows that sexual assault was weaponized by assaulters to serve vindictive purposes, and it was also used as a form of social sanction against “offenders.” Another significant finding is the fact that both men and women are vulnerable to sexual assault. Also, perpetrators could be either male or female and strangers or non‐strangers. We further observed that abusers are known to victims and sometimes family members. To perpetrate their act, abusers tend to use different forms of violence such as physical and/or psychological assault to subdue victims. These findings are grouped under different themes and discussed further.

### Awareness, knowledge, and perception of DFSA

One study revealed a high level of awareness about date rape (68.9%) and the psychoactive substances used to enable date rape among undergraduate students [15]. Sources of information about date rape were reported to be diverse in the study, and they included friends, TV, book, newspaper, radio, and others. Sedatives and alcohol were the most reported substances believed to be used for date rape. Substances used included hard drugs (including cocaine, heroin, morphine, and cannabis sativa), ethanol, methamphetamine, methaqualone, diphenhydramine, aphrodisiacs, and anesthetic injections [15].

However, knowledge of the dangers of date rape to health was poor among the respondents, as half of them did not understand the physical and psychological risks of date rape to their victim's health [15]. For those who had good knowledge of the phenomenon, age reportedly did not affect good knowledge of date rape, whereas religion was not significantly associated with the level of knowledge [15]. One of the studies revealed perceptions that are supportive of date rape [15]. Most of the participants in the study did not perceive date rape to be as serious as stranger rape. Most of the respondents observed that every woman was susceptible to date rape at least one time before marriage [15]. Some of the factors influencing these perceptions are the extent of knowledge, and religion as it was more common among Christians [15].

### Incidence and prevalence of DFSA

The occurrence of sexual assault is reported to be rising, according to one of the studies, whereas many cases were neither reported nor documented [14]. Officials of nongovernmental organizations (NGOs) in Nigeria mentioned an increasing rate of reportage of sexual assault, especially those perpetrated by familial abusers (incest and marital rape). Furthermore, some (36.8%) survivors experienced multiple or repeated sexual assault, that is, before and during the COVID‐19 lockdown, whereas others had the experience for the first time [14]. Another study in Nigeria reported a prevalence of 11.8% as 72 out of 610 female undergraduate students interviewed had experienced date rape [15]. In another study, most incidents took place in the late evening at the home of the assailant(s) (*n* = 62, 58%), and most assailants were acquaintances to the victims (*n* = 66, 62%). The most commonly used substance was ethanol in 72 patients (67%), with drugs other than ethanol being detected in 60 patients (56%) [16].

### Factors influencing DFSA

Three studies revealed some evidence of victim blaming as reasons shared by victims and non‐victims of rape interviewed [8, 14, 16]. Most people seemingly faulted survivors of rape for their assault. Survivors of rape believed that they were assaulted for different motives, such as retribution by women who experienced violence from other men, rape used as a form of punishment/corrective action for such acts as dressing like a girl/victim's personal grooming, being gay, for initiation into a gang [8]. Other reasons mentioned are walking alone at night [8, 14] and violating the curfew law [14].

Views of non‐victims on persons at risk of date rape revealed that wearing seductive dresses, demanding material things with no intention of returning the favor with sex are believed to be major risk factors for date rape [15]. Other vulnerable persons are partygoers, girls who are too trusting, girls who are too free with intimate partners, girls cheating on their partners, and others [15].

A major social factor found to be enabling DFSA is the enforcement of law and order during COVID‐19 outbreak. In Nigeria, COVID‐19 outbreak created conditions that enabled the first experience of sexual assault by the majority (63.2%) of DFSA survivors [14]. Likewise, the circumstances (such as movement restrictions, loss of income and livelihood, and nationwide lockdown) created by the outbreak was also associated with all cases of sexual assault experienced by survivors (including multiple assaults by the same perpetrator). Other related factors that empowered perpetrators and increased female exposure to sexual assault are inadequate information about movement restrictions, high rate of insecurity, and violent conduct of law enforcement agents [14].

This review shows that cultural or dominant beliefs about rape could be major enablers. However, only one of the three studies documented beliefs associated with rape [15]. The reported beliefs or perceptions about sexual assault are somewhat supportive of or promote date rape. For instance, more than two thirds of female university students (66.8%) held beliefs that date rape was common [15]. They also remarked that actual rape is perpetrated by non‐intimate partners only, as rape by an intimate partner is not serious. The belief that reporting an incidence of date rape is worse than rape was also common (65.3%) in the study [15].

### Common types of rape

The review shows little or no difference in the types of rape or sexual assault experienced by men and women. Comparatively, gang rape was common in South Africa [8, 16]. Stranger rape (52.7%) is also more prevalent than non‐stranger rape (41.3%) in Nigeria. Forms of non‐stranger rape reported are marital rape (10.5%), date rape (15.8%), incest (10.5%), and acquaintance rape (10.5%). Some of the survivors were gang‐raped [14, 16]. The forms of rape reported amongst South African men are classified into acquaintance or non‐stranger rape, stranger rape; armed rape; homophobic rape; gang rape (such as corrective‐gang rape, drug‐facilitated gang rape, pack‐hunting rape, women retributive rape, or women vengeance) [8, 16]. Others are prison rape (e.g., transactional rape and gang initiation rape) [8].

### Patterns of DFSA and characteristics of assaulters

Our review shows that both men and women were perpetrators of sexual assault. In South Africa, survivors identified both sexes as responsible for their abuse [8, 16], whereas only men were reported as assailants in Nigeria [14]. The majority of rape survivors (12/19) had never experienced sexual assault before the case reported in the study. Among those who had previous experience (7/19) of sexual assault, (4/7) claimed the same abuser perpetrated the crime [14]. Repeated sexual assault is perpetrated mostly by non‐strangers [14].

### DFSA survivors’ relationship to abusers

The studies revealed that sexual violations could be perpetrated by strangers and non‐strangers. Female students believed that male strangers and boyfriends often perpetrate date rape [15]. This is followed by male acquittance, man‐friend, and lesbians. Others are non‐strangers (fathers, siblings) and any person who is mentally unstable [15, 16]. In contrast, the majority of female survivors in Nigeria were reportedly violated by strangers (52.6% including police officers) [14]. Also mentioned are marital partners (10.5%), intimate partners (15.8%), biological fathers (5.3%), step‐fathers (5.3%), a family friend (10.5%), and neighbor (5.3%) [14]. Equally, in South Africa, male survivors mentioned uncles, friends, neighbors, and strangers as violators [8].

### Forms of violence accompanying DFSA

In Nigeria, sexual assault is found to be commonly perpetrated along with physical assault in different forms such as minor and severe beating, being held forcefully, drugging, gagging, having clothes ripped off, gang‐raping, as well as the use of different forms of weapons [14]. Likewise, beating, drugging, and insertion of sharp objects in anus are some of the assaults suffered by survivors of DFSA in South Africa [8, 16]. Furthermore, sexual assaulters used emotional or psychological torture, intimidation, hostility [14], threats of physical violence [8, 16] as well as threats of infecting the victims with a disease [8] in the course of and following the assault.

### Reportage of DFSA

A study in in Cape Town, South Africa [16] focused on DFSA survivors who reported to the Victoria Hospital Clinical Forensic Unit. This suggested care‐seeking by the victims after the heinous act. The study did not suggest improved reportage because the authors observed only 107 survivors within about 3 years. All survivors who reported the incidents in Nigeria did so at an NGO and not directly to a law enforcement agency due to distrust and lack of confidence in the police force. Disappointingly, some survivors fingered police officers as perpetrators of the rape and did not see any point in reporting the crime to the same people [14]. The severity of the physical assault inflicted and the extent of injuries sustained by survivors were the main factors believed to have motivated them to report their assault. Furthermore, rape reportage is a function of accessibility of police and support services, the difficulty of escaping from the abuser, the assaulter's social status, and fear of COVID‐19 infection [14]. Other factors are fear of stigmatization [14], and a culture of victim blaming [14, 15].

## DISCUSSION

Among Nigerians, the patterns, nature, and factors responsible for the change in sex crimes during COVID‐19‐induced lockdown in Nigeria were assessed [14], whereas among survivors in South Africa [8, 16], the context of sexual assault and characteristics of DFSA were evaluated. Considering the experiences of rape victims [14] as well as reports by other authors [17, 18, 19], control strategies for such emergency occurrences as COVID‐19, specifically the lockdown and its enforcement, should painstakingly consider the vulnerable groups. Given that women and girls have always been victims of rape and other violence, they deserve extra protection from the state. The most fundamental strategies to control sexual assault during emergency periods include the provision of functional helplines, shelters, and dedicated response teams.

Among Nigerian students, knowledge and perceptions of a specific type of rape, date rape, was investigated [15]. Based on the study design, research focus, and sample size, only the findings of three publications conducted among rape survivors [8, 14, 16] are somewhat similar and comparable. The findings of the study among university students only complement the others. The South African studies recruited all participants from the clinic [8, 16]. Encouraging survivors of DFSA and other forms of sexual assault to report first at a medical center is a significant measure for preventing infection and getting the right medical assistance that will help document the incidence as well as get the forensic evidence that will be indispensable to getting rape perpetrators convicted.

Although in the reviewed studies, the rape of men was only reported among South Africans, it is not to disregard the fact that both men and women could be victims of rape in any country. In Nigeria, several cases of men being raped are unreported [20], perhaps because men and boys were not until recently recognized as legal victims of rape because Nigeria in 2020 removed gender restrictions in the offence of rape and other related matters [14]. When sexual assaults happen to males, they are disproportionately more underreported than females. The most common dimension of the heinous act against men includes anal and oral rape. The problem is compounded because homosexuality is criminalized in up to 60% of African countries [21]. More so, a common African stereotype relates that men should be sexually domineering‐when they are violated; they are perceived as “less manly” or homosexual [22].

Therefore, in male rape, silence rules as a form of face‐saving, pride retention, and avoidance of stigmatization. When it is drug‐facilitated, for instance, by a woman or group, it is highly culturally demeaning. Such possibility of social denigration keeps many men in perpetual silence for life. The experience of secondary victimization in the form of victim blame and other adverse reactions is more intense for male rape victims, mostly due to (culturally) normative concerns [23]. More attention, although deserving, over the years has focused on female rape [24, 25, 26], with men as the major predators‐when the “predator” is preyed on, it is a “complicated” event not easily conceivable by many [27]. To find some reporting to the clinic in South Africa is highly commendable and a recommended best practice to access appropriate care.

In contrast, only some rape survivors in Nigeria were recruited through the NGO, whereas others were recruited in the community. The majority of complainants sustained physical onslaughts after being sexually assaulted, hence the reportage [14]. Evidence shows that some survivors were violated by police officers on patrol. Thus, the reason for not reporting to the police as rape survivors had more confidence in the NGOs than the security agencies. Unfortunately, unprofessional conduct and indiscipline acts such as those reported in one of the reviewed publications [14] are documented by other studies [28, 29, 30]. Unfortunately, the ease of sexual assault is facilitated when the perpetrator is armed, as in the case of security or armed personnel, which has also been documented in the form of opportunistic illegal conduct [31, 32] or in conflict settings [33, 34, 35].

Perceptions of date rape as documented among undergraduate students and experiences shared by rape survivors in Nigeria and South Africa suggest the pervasiveness of rape victim blaming. Furthermore, both studies from Nigeria reaffirm that rape stigma is very much entrenched in many countries, and this may prevent survivors from reporting their assault and from getting the deserved justice [36]. The occurrence of heterosexual female rape has been attributed to unequal gender power relations [37, 38]; hence, most male perpetrators often feel entitled to sex from their girlfriends or assume they should enjoy it despite the absence of consent‐therefore heinously legitimizing the use of force [39, 40]. Unfortunately, gender inequality gap is still wide in Africa [41, 42] and the engendered circumstances might be exploited in the perpetuation of sexual violence against women. The worst scenario is that the victims suffer from additional re‐victimization, including rape stigma, which is widespread [43, 44]. Rape stigma often leads to self‐blame, unconstructive and negative normative judgment and lack of sympathy or empathy toward survivors [45, 46].

As such, typical DFSA cases appear to be opportunistic in nature and mostly involve women in their mid‐20s and an acquaintance as the culprit [4, 16]. However, disaggregating DFSA as a specific sexual assault requires some logistics, including forensic and toxicological investigations [47]. The South African studies [8, 16] documented the complexity of the investigation of DFSA. This complexity might be responsible for the low declaration of some cases as DFSA. Many countries could also suffer from inadequate forensic know‐how in the investigation of DFSA. Hence, most cases are treated as mere sexual assault. The dearth of scientific literature on DFSA among Africans compared to rape or sexual assault at large upholds our observation. Hence, the development of infrastructure to support systematic toxicological analyses and services to assist in investigating and understanding these intricate cases has been advocated [16]. This is in concordance with the recommendation of the United Kingdom's Advisory Council on The Misuse of Drugs (ACMD). Evidence gathering through forensic examination was one of the major steps toward sexual assault control [48].

Furthermore, the rising epidemic of substance abuse among African youths suggests that DFSA will continue to be a public health challenge. To stem DFSA risks, strategies such as minding drinks when on a date, avoiding drinks from unknown sources or persons, and provision of “early evidence kits” at emergency units of hospitals are advised [48]. Likewise important is the strengthening of law enforcement to stop the marketing and use of illicit substances.

## CONCLUSION

DFSA is a significant dimension of gender‐based violence. Sedatives and alcohol are the most reported substances used in such rape. Other substances are hard drugs, aphrodisiacs, spraying chemicals/aerosols into the air, and anesthetic injection. Unfortunately, most respondents still held perceptions supportive of date rape by blaming the victims, especially their “seductive” dresses. There is a definite research gap in this area. There is a need to interrogate how and what drugs are used in different countries for the purpose of control. Only four studies in over 54 African countries are quite low to inform a continent‐wide intervention purposes. However, regulations around hard drugs and controlled sedatives must be strengthened to prevent unprescribed use. Culturally, there should be reorientation against hegemonic masculinity due to unequal gender hierarchy, control of women, and objectification of women. Nursing and pastoral care interventions for victims of sexual assault should always consider that males are also potential victims of sexual assault, without downplaying that men are often the perpetrators of sexual violence against females and children. Irrespective of gender, victim care responses should address blame and rape stigma. Cultural reorientation or shift should focus on sexual rights and consent in sexual relationships.

## AUTHOR CONTRIBUTIONS

*Study conceptualization*: Jimoh Amzat and Kehinde Kazeem Kanmodi. *Study protocol design*: Kehinde Kazeem Kanmodi. *Data collection*: Kehinde Kazeem Kanmodi and Eyinade Adeduntan Egbedina. *Data analysis*: Jimoh Amzat and Kafayat Aminu. *Manuscript drafting*: Jimoh Amzat, Kehinde Kazeem Kanmodi, and Kafayat Aminu. *Review of drafts*: Jimoh Amzat, Kehinde Kazeem Kanmodi, and Kafayat Aminu. *Acceptance of the final draft*: Jimoh Amzat, Kehinde Kazeem Kanmodi, Kafayat Aminu, and Eyinade Adeduntan Egbedina. All authors have read and agreed to the published version of the manuscript.

## FUNDING INFORMATION

This study was self‐funded.

## CONFLICT OF INTEREST STATEMENT

Authors have no conflicts of interest to declare.

## PATIENT CONSENT STATEMENT

Not applicable.

## ETHICS STATEMENT

Not applicable. This study is a scoping review.

## TRANSPARENCY STATEMENT

The corresponding author—Kehinde Kazeem Kanmodi—affirms that this manuscript is an honest, accurate, and transparent account of the study being reported; that no important aspects of the study have been omitted; and that any discrepancies from the study as planned (and, if relevant, registered) have been explained.

## ETHICAL CONSIDERATIONS

Not applicable. This study did not collect data from human or animal subjects but an open research repository.

## Supporting information

Supporting Information

## Data Availability

Data sharing is not applicable to this article as no new data were created or analyzed in this study. All authors have read and approved the final version of the manuscript. Kehinde Kazeem Kanmodi had full access to all of the data in this study and takes complete responsibility for the integrity of the data and the accuracy of the data analysis.
